# Correlation between Glycated Hemoglobin and Triglyceride Level in Type 2 Diabetes Mellitus

**DOI:** 10.7759/cureus.1347

**Published:** 2017-06-13

**Authors:** Syeda Naqvi, Shabnam Naveed, Zeeshan Ali, Syed Masroor Ahmad, Raad Asadullah Khan, Honey Raj, Shoaib Shariff, Chintan Rupareliya, Fatima Zahra, Saba Khan

**Affiliations:** 1 Jinnah Postgraduate Medical Centre, Jinnah Sindh Medical University (SMC); 2 Internal Medicine, Jinnah Sindh Medical University (SMC); 3 Jinnah Postgraduate Medical Centre, Baqai Medical College; 4 USMLE, Medsmarter Test Prep; 5 Department of Neurology, University of Missouri, Columbia, Missouri; 6 Medicine, Liaquat Medical and Dental College

**Keywords:** diabetes, diabetes education, triglyceride, lipid, hba1c, dyslipidemia, cardiovascular event, smoking, hypertension, fasting plasma glucose

## Abstract

**Context:**

Dyslipidemia is quite prevalent in non-insulin dependent diabetes mellitus. Maintaining tight glycemic along with lipid control plays an essential role in preventing micro- and macro-vascular complications associated with diabetes.

**Purpose:**

The main purpose of the study was to highlight the relationship between glycosylated hemoglobin (HbA1c) and triglyceride levels. This may in turn help in predicting the triglyceride status of type 2 diabetics and therefore identifying patients at increased risk from cardiovascular events. Hypertriglyceridemia is one of the common risk factors for coronary artery disease in type 2 diabetes mellitus (DM). Careful monitoring of the blood glucose level can be used to predict lipid status and can prevent most of the complications associated with the disease.

**Method:**

This is a cross-sectional study using data collected from the outpatient diabetic clinic of Jinnah Postgraduate Medical Centre (JPMC) Karachi, Pakistan. Patients of age 18 years and above were recruited from the clinic. A total of consenting 509 patients of type 2 diabetes mellitus were enrolled over a period of 11 months.

For statistical analysis, SPSS Statistics for Windows, Version 17.0 ( IBM Corp, Armonk, New York) was used and Chi-square and Pearson’s correlation coefficient was used to find the association between triglyceride and HbA1c. The HbA1c was dichotomized into four groups on the basis of cut-off. Chi-square was used for association between HbA1c with various cut-off values and high triglyceride levels. Odds-ratio and its 95% confidence interval were calculated to estimate the level of risk between high triglyceride levels and HbA1c groups. The p-value < 0.05 was considered statistically significant for all the tests applied for significance.

**Result:**

The association of high triglyceride was evaluated in four different groups of HbA1c, with a cut-off seven, eight, nine and 10 respectively. With HbA1c cut-off value of 7%, 74% patients had high triglycerides and showed a significant association with high triglyceride levels at p < 0.001 and odds ratio was 2.038 (95% confidence interval: 1.397 – 2.972). Logistic regression models were adjusted for demographic factors (age, race, gender), lifestyle factors (smoking, body mass index, lifestyle) and health status factors (blood pressure, physician-rated health status).

**Conclusion:**

After adjusting for relevant covariates, glycated hemoglobin was positively correlated with high triglyceride. Hence, HbA1c can be an indicator of triglyceride level and can be one of the predictors of cardiovascular risk factors in type 2 diabetes mellitus.

## Introduction

Type 2 diabetes mellitus is one of the prevalent diseases increasing health burden in both developed and underdeveloped countries. Prevalence of diabetes is noted to be higher in Asians (people from Pakistan, India, and China) as compared to Caucasians [1-2]. The significant factors responsible for this outstanding prevalence in Asians as compared to Caucasians are the sedentary lifestyle changes, rapid urbanization & adoption of industrialized food culture which leads to obesity and insulin resistance. In Pakistan, a recent study showed that the prevalence of non-communicable diseases like type 2 diabetes mellitus has doubled as compared to data collected in 2004. The health burden of diabetes in some of the semi-urban area was found to be 14.6% [3]. 

Diabetes Mellitus is an array of metabolic dysfunction secondary to decreased insulin secretion or insulin resistance. The common threat of this disease is poor glycemic control predisposing to micro- and macro-vascular complications. Microvascular complications include neuropathy, retinopathy, and nephropathy. Macrovascular complications are coronary artery and peripheral artery disease. Type 2 diabetes is linked with high cardiovascular morbidity and mortality. Cardiovascular risk in diabetes is determined by dyslipidemia and hypertension [4-5].

Dyslipidemia is one of the common condition associated with a poor glycemic control in type 2 DM. The pathogenesis of dyslipidemia in type 2 DM is a decrease in activity of lipoprotein lipase due to insulin deficiency or resistance. Under the action of insulin, enzyme lipoprotein lipase metabolizes lipids in a healthy individual. In type 2 DM, the relative insulin deficiency and decreased adiponectin causes decrease lipoprotein lipase activity resulting in high levels of low-density lipoprotein (LDL), triglyceride and low levels of high-density lipoprotein (HDL). Qualitative defects in LDL are also seen in type 2 diabetes including atherogenic, glycated or oxidized LDL further amplifying the risk of atherogenesis. [6-7]. 

Dyslipidemias is one of the modifiable risk factors for coronary artery disease in type 2 diabetes. Atherogenic or diabetic dyslipidemia is defined by a profile of low- and high-density lipoprotein and high triglycerides. It is an independent predictor of coronary artery disease or silent myocardial ischemia [3,8]. High triglycerides can be dictated by many factors including genetic or acquired [9]. To rule out other causes, we only include patients without any familial dyslipidemia or history of alcohol intake. As these two factors also play a role in increasing triglycerides as compared to polygenic etiologies like obesity, insulin resistance or diabetes mellitus [10-11]. 

The literature review was evident that hypertriglyceridemia is linked to high glucose levels and increased risk of type 2 diabetes [12-13]. In our study, correlation of HbA1c with high triglycerides signifies HbA1c as a direct marker of hypertriglyceridemia and an indirect marker of risk assessment of coronary artery disease. It is important to understand the concept of insulin resistance and dyslipidemia predisposing to atherosclerosis [14-15]. Cholesterol lowering through secondary prevention by lifestyle changes or statin therapy has tremendously improved cardiac outcome in diabetes [5].

## Materials and methods

In this cross-sectional analytical study, we included 509 consenting patients of either gender from outpatient diabetes clinic Medical Unit-III, ward 7 Jinnah Postgraduate Medical Centre, suffering from type 2 diabetes. Our age limit was 18 and above. Demographic information was collected from all patients included age, gender, body mass index (BMI), active tobacco use, active alcohol use, hypertension status, employment status and marital status. Relevant medical history including the presence of the type of diabetes, hypertension, pulmonary comorbidities, cardiac comorbidities (i.e. arrhythmia, myocardial infarction and coronary artery disease), thyroid abnormalities and the presence of familial dyslipidemia was also obtained.

Our exclusion criteria were patients with missing data, taking statins or suffering from renal, hepatic, cardiac or thyroid diseases, and familial hypercholesterolemia. Venous samples of patients were collected after 12-hour fasting for measurement of triglyceride levels and glycated hemoglobin (HbA1c) for a period of 11 months. Glycosylated hemoglobin (HbA1c) was determined by a high-performance liquid chromatographic (HPLC) method. Serum triglycerides were estimated by enzymatic colorimetric method using glycerol kinase.

Data analysis was done through SPSS version -17 (IBM Corp, Armonk, New York). Continuous variables like age, lipid function test, HbA1c, and duration of diabetes were described, mean while categorical variables like gender, risk factor history like smoking, comorbidities like peripheral arterial disease and hypertension and lifestyle were presented in frequencies and percentages. Pearson correlation test (r) was used to evaluate the relationship between HbA1c and triglycerides. Chi-square was used for association between HbA1c with various cut-off values and high triglyceride levels. Odds-ratio & its 95% confidence interval was calculated to estimate the level of risk between high triglyceride levels and HbA1c groups. The p-value < 0.05 was considered statistically significant for all the tests applied for significance.

## Results

A total of 509 subjects with type 2 Diabetes fulfilled the inclusion criteria. Of that 41.9 % were males and 57.9% were females. Moreover, 67.4% were less than 50 years of age. Mean age was 48.81 ± 10.23 years and their body mass index based on their body weight at the time of enrollment in the study was 26.97 ± 2.74kg/m2. The HbA1c level was used as a marker of glycemic control. In 67.8% (n=345) subjects HbA1c was more than 7%. Prevalence of high triglyceride (triglyceride > 150) was 58.2 % (n=296). High levels of LDL (LDL more than 100) was found in 66% (n=336) and 81.7% subjects had a sedentary lifestyle.

Glycated hemoglobin is dichotomized into four groups i.e. seven, eight, nine and 10 respectively. With a cutoff of 7%, 74% (n=220) had high triglycerides(TG > 150) and showed a significant association at p <0.001 and odds ratio was calculated to be 2.038 (95% C.I.: 1.397 – 2.972). With a cut-off HbA1c as 8%, 48% (n=142) had high triglycerides and showed a significant association at p <0.001 and odds ratio was calculated to be 2.26 (95% C.I. : 1.56 – 3.26). Utilizing the same dichotomization scheme, a cut-off HbA1c as 9%, 68.6% (n=203) had high triglycerides and showed a significant correlation at p <0.001 and odds ratio was 2.69 (95% C.I. : 1.397 – 2.972). With a cut-off HbA1c as 10%, 78.4% (n=232) had high triglycerides and showed a significant correlation at p <0.001 and odds ratio was calculated to be 2.66 (95% C.I. : 1.55 – 4.55) as shown in (Table 1).

**Table 1:** Table showing odds ratio

HbA1c	HbA1c ≤7 or>7	HbA1c ≤8 or>8	HbA1c ≤9 or>9	HbA1c ≤10 or>10
Odds ratio (Confidence interval)	2.03 (C.I=1.39-2.97)	2.26 (C.I=1.56-3.26)	2.69 (C.I=1.71-4.23)	2.66 (C.I=1.55-4.55)
High Triglycerides (TG>150)	74.3% (n=220)	52% (n=154)	31.4% (n=296)	21.6% (n=296)

Selected characteristics were determined in four groups of HbA1c and their mean value is tabulated in (Tables 2-3).

**Table 2:** Table showing selected characteristics of subjects by glycosylated hemoglobin (HbA1c) groups

HbA1c Group (percentage)	HbA1c less than or equal to 7	HbA1c=8	HbA1c=9	HbA1c=10	HbA1c>11
Females	59%	55%	56%	69%	56%
Hypertension	38%	53%	55%	23%	47%
Smokers	46%	52%	26%	10%	39%
Peripheral artery disease	40%	75%	33%	11%	31%
Sedentary lifestyle	83%	93%	68%	79%	77%

**Table 3:** Table showing selected variables of subjects by glycosylated hemoglobin (HbA1c) groups

HbA1c Group (mean±standard deviation)	HbA1c less than or equal to 7	HbA1c=8	HbA1c=9	HbA1c=10
Age in years	49.10 ± 9.57	47.96 ± 9.61	48.44 ± 11.19	51.74 ± 9.44
Serum cholesterol	169.37 ± 42.67	186.23 ± 40.68	179.74 ± 57.89	189.44 ± 43.62
Serum triglycerides	173.92 ± 84.13	183.07 ± 98.36	207.33 ± 89.83	218.62 ± 105.01
Serum low density lipoprotein (LDL)	109.71 ± 49.07	151.01 ± 52.83	152.61 ± 51.16	168.26 ± 78.03
Serum high density lipoprotein (HDL)	33.97 ± 5.31	37.08 ± 4.99	35.33 ± 6.20	35.74 ± 6.74
Duration of diabetes in years	6.19 ± 4.18	7.88 ± 3.99	6.78 ± 4.31	5.41 ± 3.01

**Figure 1:**
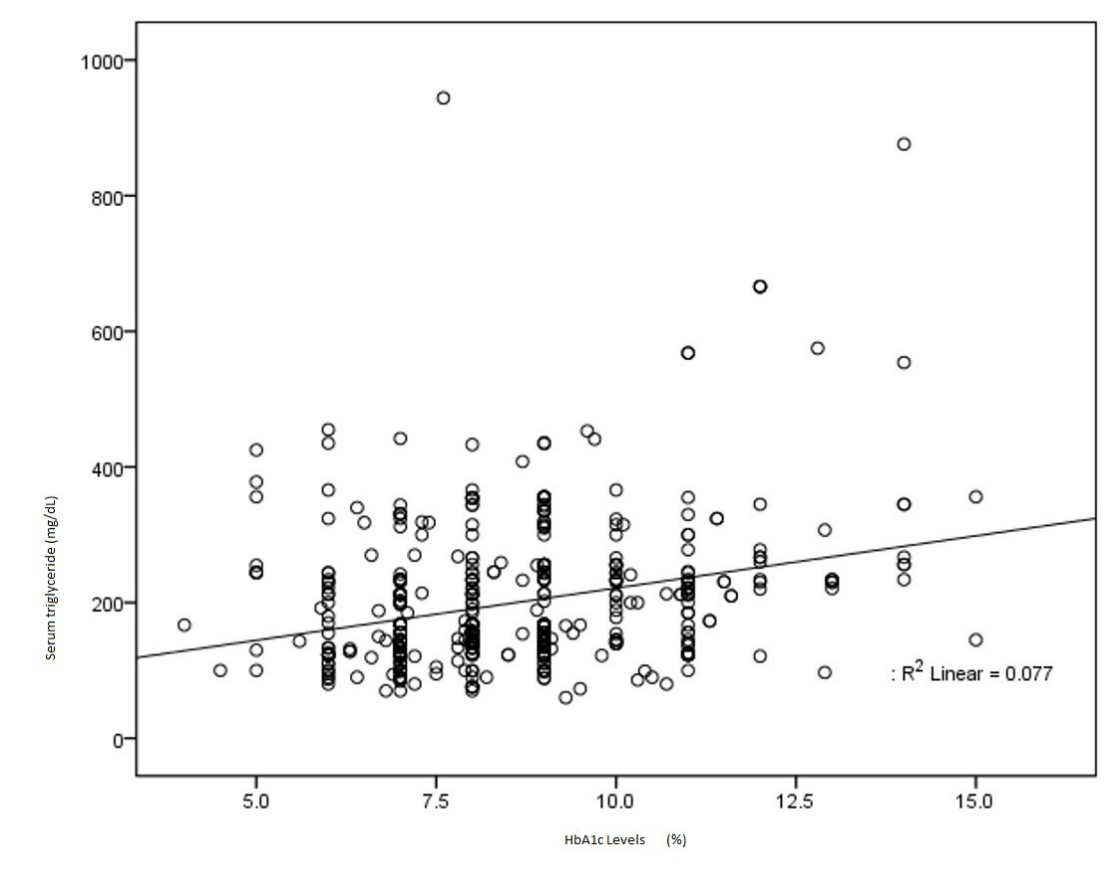
Scattered plot showing correlation between glycosylated hemoglobin (HbA1c) and triglyceride levels

Pearson’s correlation coefficients were applied to establish the correlations between glycated hemoglobin and triglyceride. Results of the univariate analysis showed that HbA1c is significantly correlated with high triglyceride levels (r=0.278, p value< 0.0001). Along with glycemic control, HbA1c can also be used as a marker of dyslipidemia especially hypertriglyceridemia. A scattered plot has shown the correlation between glycemic control and high triglyceride level in (Figure 1).

## Discussion

Diabetes is a multifactorial disorder having a wide range of lipid abnormalities. In type 2 diabetes mellitus, there is an increased incidence of hypertriglyceridemia as compared to other lipid abnormalities [16-17]. This study evaluated the correlation between glycated hemoglobin (HbA1c) and triglycerides level and the results showed that there is a significant correlation between high HbA1c and high triglyceride. This may in turn help in predicting the triglyceride status of type 2 diabetics from the degree of glycemic control and therefore identifying patients at increased risk from cardiovascular events [18-19].

Lebovitz suggested that there is a lipotoxic mechanism by triglyceride which interferes with gastric or neural pathway which regulates glycemic control [19]. In most of the studies, there is a correlation found between glycemic control and dyslipidemia [20]. In a recent study, it was evident that there was a positive correlation between HbA1c and high triglycerides and HbA1c can be used as a potent marker for dyslipidemia and mitigate the macro- and micro-vascular complications of disease [21].

Diabetes is an independent risk factor for developing cardiovascular risk. Cardiovascular events are also the most common cause of death in diabetes [22-23]. Gluco-centric medications might help in improving glycemic control but their role in preventing cardiovascular disease is limited. According to the records mandated by American Association of Clinical Endocrinology (AACE). In 2016, approximately 660,000 United States residents will have a new coronary event as myocardial infarction or atherosclerotic cardiovascular event (ASCVD) [24]. It further mentions dyslipidemia as major risk factor for ASCVD. Increasing evidence points towards insulin resistance lead to hypertriglyceridemia and increased low-density lipoprotein (LDL) and decrease in high-density lipoprotein (HDL), as also an important risk factor for developing ASCVD and peripheral artery disease. According to our study, high HbA1c (cut-off of 9%) increased the risk of hypertriglyceridemia by 2.69 (OR=1.71-4.23, p <0.001). In other words, poor glycemic control would increase the risk of hypertriglyceridemia by 2.69% on average. This suggests an increased risk of atherogenicity due to dyslipidemia associated with poor diabetes control [25].

The main purpose of this study was to increase the awareness among physician about the relationship between hypertriglyceridemia and increasing HbA1c in type 2 diabetes so that physicians should be vigilant in monitoring fasting triglyceride levels every six months in type 2 diabetic patients with increased HbA1c levels. According to AACE and American College of Endocrine (ACE) Board, 10-year risk for ASCVD is high in patients with both diabetes and hyperlipidemia. It is recommended that fitness therapy should be advised for diabetes patients that are exercise programs that include 30 minutes of moderate-intensity physical activity (consuming four-seven kcal/minute), four to six times weekly with an expenditure of at least 200 kcal [23]. Lifestyle modification and regular exercise along with statin and fenofibrate play a vital role in treating diabetic dyslipidemia [24].

The role of statin in combination to fibrates in treating hypertriglyceridemia is still unclear [26]. More randomized control trial study is needed to clarify statin's role in the treatment of hypertriglyceridemia. However, it is evident that statin reduces the risk of cardiovascular mortality caused by diabetic dyslipidemia which is most commonly caused by hypertriglyceridemia [27].

## Conclusions

In this study, there is a significant correlation between glycemic control and triglyceride levels in patients with type 2 diabetes in this population. Familiarity with this concept help to diagnose lipid abnormalities in patients with poor glycemic control and preventing cardiovascular events in the high-risk patient.
